# *CACNA1A* Gene Variants in Eight Chinese Patients With a Wide Range of Phenotypes

**DOI:** 10.3389/fped.2020.577544

**Published:** 2020-12-03

**Authors:** Linxia Zhang, Yongxin Wen, Qingping Zhang, Yan Chen, Jiaping Wang, Kaili Shi, Lijun Du, Xinhua Bao

**Affiliations:** ^1^Department of Pediatric, Peking University First Hospital, Beijing, China; ^2^Department of Neurology, Children's Hospital of Shanxi, Taiyuan, China

**Keywords:** *CACNA1A* gene, sporadic hemiplegic migraine type 1, episodic ataxia type 2, genotype-phenotype correlations, developmental delay

## Abstract

**Background:** The *CACNA1A* gene encodes the voltage-dependent P/Q-type calcium channel subunit alpha-1A, which is widely expressed throughout the CNS. The biological roles of the P/Q channel are diverse and the phenotypic spectrum caused by *CACNA1A* mutations is wide. The aim of this study is to demonstrate its phenotypic diversity and analyze the genotype-phenotype correlations in a cohort of Chinese patients.

**Methods:** Patients with hemiplegic migraine, cerebellar ataxia, developmental delay, or epilepsy without known causes were tested by trios whole-exome sequencing. Patients with pathogenic *CACNA1A* gene variants were recruited. The clinical information of the patients was collected, and the association between the genotype and the phenotype was investigated.

**Results:** In total, eight patients (six females and two males) were found to have *CACNA1A* gene variants. All the variants were *de novo* including six missense variants and one frameshift variant. Four *de novo* missense variants were found in five patients located in the S4, S5, or S6 transmembrane segments of Domain II and III (p.R1352Q, p.G701V, p.A713T, p.V1393M). All of them were correlated with severe phenotypes, including three with sporadic hemiplegic migraine type 1 and epilepsy, and two with developmental and epileptic encephalopathy. The other two missense variants, p.Y62C and p.F1814L, located in the cytoplasmic side of the N-terminus and C-terminus, respectively. The variant p.Y62C was associated with severe hemiconvulsion-hemiplegia-epilepsy syndrome, and p.F1814L was associated with relatively mild phenotypes. All the missense variants were speculated as gain-of-function (GOF) mutations. The only frameshift variant, p.Q681Rfs^*^100, a lose-of-function (LOF) mutation, was found in a patient with episodic ataxia type 2. Meanwhile, all the patients had developmental delay ranging from mild to severe, as well as cerebellar ataxia including one with congenital ataxia, one with episodic ataxia, and six with non-progressive ataxia.

**Conclusions:** *CACNA1A* variants could lead to a wide spectrum of neurological disorders including epileptic or non-epileptic paroxysmal events, cerebellar ataxia, and developmental delay. The variants could be both GOF and LOF mutations. There appeared to be some correlations between genotypes and phenotypes.

## Introduction

The *CACNA1A* gene (MIM^*^601011) is located at 19p13.13 and encodes the subunit alpha-1A of the voltage-dependent P/Q-type calcium channel (Ca_V_2.1 channel) (1). The P/Q channel is widely expressed throughout the central nervous system, with diverse biological roles. Traditionally, heterozygous pathogenic variants in *CACNA1A* may lead to several different phenotypes, including familial/sporadic hemiplegic migraine type 1 (FHM1/SHM1, MIM#141500), familial hemiplegic migraine with progressive cerebellar ataxia (MIM#141500), episodic ataxia type 2 (EA2, MIM#108500), and spinocerebellar ataxia type 6 (SCA6, MIM#183086). Furthermore, *CACNA1A* mutations have recently been identified in subjects with early infantile epileptic encephalopathy 42 (EIEE42, MIM#617106), developmental delay, intellectual impairment, autism, and episodic events, such as benign paroxysmal torticollis (BPT), benign paroxysmal vertigo (BPV), benign paroxysmal tonic upgaze (PTU) (2, 3), which give us the impression that *CACNA1A* variants are associated with a wide phenotypic spectrum.

However, most of the research are case reports, and very few reports have published the relationship between the genotype and phenotype of *CACNA1A*-related disorders. Herein, we describe eight Chinese children with *CACNA1A* variants, in an attempt to demonstrate the diversity of phenotypes and the genotype-phenotype correlations.

## Patients and Methods

### Patients

Two hundred and eighty patients who presented to the Department of Pediatrics, Peking University First Hospital between January 2015 and January 2020 with hemiplegic migraine (HM), cerebellar ataxia, developmental delay, or epilepsy without known causes assessed by the workup of biochemistry, metabolism, imaging, immunology, and CNS infection were recruited. This study was approved by the hospital ethics committee [No. (2005) 004]. Written informed consent for clinical data collection and genetic studies were obtained from the patients' legal representatives.

### Genetic Assessment

Trios whole-exome sequencing was performed. Variants identified by next-generation sequencing were validated by Sanger sequencing. The *CACNA1A* gene variants presented in the patients were considered as deleterious according to the following criteria: they were variants leading to a stop codon or they had already been reported as disease-causing variants; or they were: (1) absent from the dpSNP, GnomeAD, and ExAc databases; (2) prediction tools, including SIFT (http://sift.jcvi. org/), PolyPhen-2 (http://genetics.bwh.harvard.edu/pph2/), and Mutation Taster (http://www.mutationtaster.org/) gave results in favor of a deleterious effect on the gene; (3) they were localized in the domain critically implicated in channel function; (4) the patient's phenotype was typical of a CACNA1A linked disease.

### Clinical Assessment

Clinical information was collected retrospectively and prospectively by face-to-face interviews with patients and their families. A structured questionnaire was specifically designed to gather information concerning gender, age at onset, age at last follow-up, perinatal events, psychomotor development, familial history, paroxysmal neurological events, neurological examination, duration of the episodes, treatments, and outcome. Magnetic resonance imaging (MRI), video electroencephalogram (Video-EEG) and standardized cognitive scales data were also collected and reviewed by an experienced pediatric neurologist. The end of the follow-up period was in April 2020.

### Genotype-Phenotype Correlations

Data of genotype (DNA variant), protein modification, and clinical information including HM, seizure, developmental delay, and intellectual disability (ID) as well as the other neurological phenotypes were collected. The variants in each protein structure regarding their locations in the transmembrane, extracellular, or cytoplasmic domains were also plotted. Then, the association between variant location and phenotype was investigated.

## Results

### *CACNA1A* Variants

**Figure 1:**
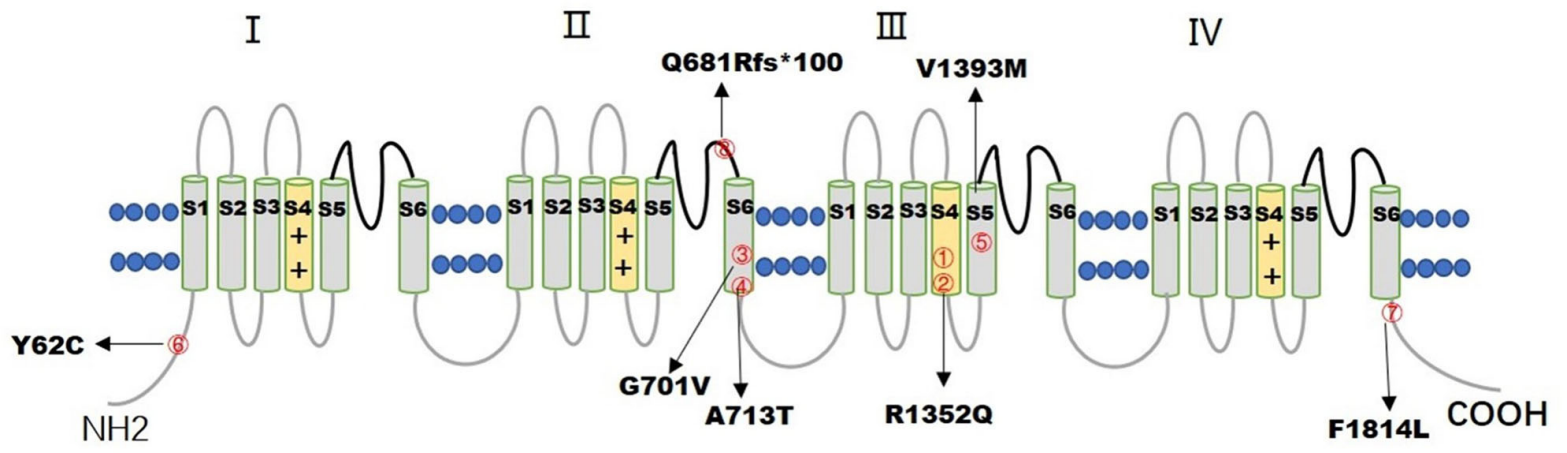
The figure illustrates the distribution of 7 *CACNA1A* variants in the Ca_V_2.1 channel. The numbers inside the red circles correspond to the number of patients.

Seven *de novo CACNA1A* gene variants were found in the eight patients (Table 1). The variants distributed in the Ca_V_2.1 channel are illustrated in Figure 1. All the detected variants were missense variants except the one in patient eight, which was a reported pathogenic frameshift variant, C.2042-2043delAG (p.Q681Rfs^*^100) (4). Patient 1 and patient 2 harbored the same missense variant, C.4055G>A (p.R1352Q), which was reported previously (2, 5–7). The missense variants in patient 4 and patient 5 were C.2137G>A (p.A713T) and C.4177G>A(p.V1393M), respectively, also reported previously (8–11). Patients 3, 6, and 7 had novel missense variants, C.2102G>T (p.G701V), C.185A>G (p.Y62C), and C.5442T>G (p.F1814L), respectively. A prediction of the mutation effect was carried out using SIFT, PolyPhen_−_2, and Mutation Taster, and all of them were shown to be disease-causing or damaging.

**Table 1:** *CACNA1A* gene variants (Genebank transcript ID: NM-000068) and the clinical characteristics.

**Patients**	**Variants**	**Domain**	**HM**	**AE**	**Cerebellar /brainstem symptoms**	**Seizures**	**Other phenotypes**	**Diagnosis**	**Previous report references**
1	C.4046G > A (p.R1352Q)	IIIS4	SHM	(+)	CA, strabismus, dysarthria, nystagmus	FS	GDD, ID	SHM1	SHM1 and CA (2, 5)
2	C.4046G > A (p.R1352Q)	IIIS4	SHM	(+)	NA, PTU, strabismus, dysarthria	FS, SE	GDD, ID	SHM1	SHM1 and seizures (6, 7)
3	C.2102G > T (p.G701V)	IIS6	SHM, N-HM	(+)	NA	FS, SE	GDD, ID	SHM1	(-)
4	C.2137G>A (p.A713T)	IIS6	(-)	(-)	NA, ST	FS, SE	GDD	DEE	LGS (9, 10) EIEE (8)
5	C.4177G>A (p.V1393M)	IIIS5	(-)	(-)	NA, ST	FS SE, FC	GDD	DEE	EE (9), LGS (11)
6	C.185A > G (p.Y62C)	N-term	(-)	(-)	NA	FS, SE, HHE	LDD	HHE	(-)
7	C.5442T > G (p.F1814L)	C-term	(-)	(-)	NA	AAS, GTCS, FC	LDD	Epilepsy	(-)
8	C.2042-2043delAG (p.Q681Rfs*100)	IIS5-IIS6	(-)	(-)	EA, nystagmus	(-)	LDD	EA2	EA2 (4, 12)

*AAS, atypical absence seizure; AE, acute encephalopathy; CA, congenital ataxia; C-term, C-terminus; DD, developmental delay; EA, episodic ataxia; EE, epileptic encephalopathy; EIEE, early infantile epileptic encephalopathy; FC, febrile convulsion; FS, focal seizures; GTCS, generalized tonic-clonic seizure; GDD, global developmental delay; HM, hemiplegic migraine; HHE, hemiconvulsion-hemiplegia-epilepsy; ID, intellectual disability; LDD, language developmental delay; LGS, Lennox-Gastaut syndrome; NA, non-progressing ataxia; N-HM, non-hemiplegic migraine; N-term, N-terminus; PTU, paroxysmal tonic upgaze; SE, status epileptics; ST, status tremor*.

### Clinical Description and Investigation

The phenotypes of eight patients, six females, and two males, with *CACNA1A* gene variants, included three patients with SHM1 manifesting as recurrent severe encephalopathy and hemiplegia (patients 1–3), two patients with developmental and epileptic encephalopathy (DEE) (patients 4 and 5), one patient with hemiconvulsion-hemiplegia-epilepsy syndrome (HHE) (patient 6), one patient with epilepsy having atypical absence and tonic-clonic seizures (patient 7), and one patient with EA2 (patient 8). All the patients had developmental delay ranging from mild to severe, and cerebellar ataxia including one with congenital ataxia, one with episodic ataxia, and six with non-progressive ataxia. The initial clinical presentation included one hemiplegic migraine (patient 3), two hypotonia and abnormal eye movement (patients 1 and 2), three focal status epilepticus (SE) (patients 4–6), one febrile convulsions (patient 7), and one episodic ataxia (patient 8). The median age of initial presentation was 14 months (ranging from 3 month to 2 year). All eight patients were born at term without perinatal or neonatal problems. All of them were sporadic cases without positive family history. The clinical manifestation, neuroimaging, EEG, and molecular genetic profile are summarized in Tables 1, 2. Detailed clinical information is provided in the following.

**Table 2:** Clinical features of the eight patients with *CACNA1A* variants.

**Clinical assessment**		**Number of affected patients/total number of patients**
Hemiplegic migraine		3/8
	With fever	3/8
	With coma	3/8
	Induced by minor head trauma	3/8
	Number of attacks >5	1/8
	Number of attacks ≤ 5	2/8
	Non-hemiplegic migraine	1/8
Cerebellar ataxia		8/8
	Congenital cerebellar ataxia	1/8
	Episodic cerebellar ataxia	1/8
	Non-progressive ataxia	6/8
Paroxysmal events	PTU	1/8
Epileptic seizures		7/8
	Focal seizures	6/8
	Atypical absent seizures	1/8
	Status epilepticus	5/8
	Heat-sensitive seizures	7/8
Developmental delay		8/8
	Global developmental delay	5/8
	Language developmental delay	3/8
Intellectual disability		3/8
Neurological examination	Abnormal	8/8
	Strabismus	2/8
	Nystagmus	2/8
	Dysarthria	2/8
	Gait ataxia	8/8
	Intention tremor	5/8
	Static tremor	2/8
	Hemiplegia	1/8
Brain MRI	Abnormal	4/8
	Cerebellar atrophy	3/8
	Cerebral edema	2/8
	Hemisphere atrophy	2/8
V-EEG	Abnormal	7/8
	Asymmetric background	4/8
	Focal discharge	4/8
	Generalized high-amplitude spike and slow waves	1/8

### Hemiplegic Migraine

Patients 1, 2, and 3 had severe hemiplegic migraines. Patient 1, a 15-year-old girl, presented with episodes of HM when she was 4 years old. The attacks were characterized by speech difficulties, headache, vomiting, and weakness, mostly on the left side, but occasionally on the right side or bilaterally, sometimes accompanied by facial nerve palsy or hemiconvulsions. It used to take 24 h to 21 day for a full recovery. The patient had experienced recurrent episodic attacks more than 10 times so far.

Patient 2, a 7-year-old boy, presented with a severe HM episode at 3 years of age after a fall from a sofa. After crying for half an hour, he began to vomit, followed by confusions, fever, left hemiplegia, and intermittent left hemiconvulsions, which lasted for about 1 h. Both the brain CT scan and cerebrospinal fluid examination were normal. He completely recovered 2 day later. At 7 years of age, he experienced a second severe attack with vomiting, headache, right hemiplegia, and lethargy. It was also triggered by mild head trauma. His temperature rose to 39.5°C the next day. He made a full recovery 10 day after admission.

Patient 3, an 8-year-old girl, had five episodic events of vomiting, headache, and hemi-clonic seizure after 2 years of age. Occasionally, hemiplegia developed after a unilateral focal seizure, and she recovered after a brief sleep within 24 h. She suffered the last episode at age 6, with the manifestation of frequent vomiting, left-side convulsion, hemiplegia on the left side, and lethargy. The hemi-clonic seizure lasted for 1 h, and her temperature rose the next day. She regained consciousness on day 3 and recovered from hemiplegia on day 14.

All three patients had recurrent severe episodes of HM characterized by coma, prolonged hemiplegia, and hemiconvulsions, which tended to be triggered by trivial head trauma, and usually progressed rapidly to coma when accompanied by fever. They all made a full recovery from each episode, and only patient 3 had complained of a headache between the attacks.

### Cerebellar/Brainstem Symptoms

All patients in the cohort presented with cerebellar ataxia. Patient 1 exhibited congenital ataxia. She had hypotonia and nystagmus at age 3 months. A brain CT scan performed at 8 months demonstrated cerebellar dysplasia. Patient 8 exhibited episodic ataxia after 2 years of age, he experienced recurrent episodes of dizziness and imbalance without altering consciousness. The episodes lasted 3 to 5 min in duration and occurred 1 to 6 times per day. It could be triggered by exertion and sweating. Sometimes, the episode was so severe that he could not walk steadily, along with vomiting. The neurological examination indicated a horizontal nystagmus and unstable tandem walking. The remaining patients presented as non-progressive ataxia of varying severity characterized by ataxic gait and intention tremors. Both patients 1 and 2 had dysarthria. No progressive ataxia were noticed clinically or on neuroimaging. In addition, abnormal eye movements and static tremors were also observed in four patients. Patient 1 developed strabismus at 3 months, patient 2 had strabismus and PTU at 4 months. Patients 4 and 5 had static tremors in the head and trunk.

### Seizures

Seven patients (patients 1–7) developed epilepsy. In which, six exhibited focal seizures, only one (patient 7) exhibited atypical absence seizures and generalized tonic-clonic seizures. Patients 1, 2, and 3 had epileptic seizures during attacks of HM with heat sensitivity. Patient 3 also had seizures between migraine attacks. Focal SE occurred in patients 2 and 3 several times. Patients 4, 5, and 6 had their first epileptic episode at age 11 months, 20 months, and 12 months, respectively, all presenting as focal SE. The frequency of seizures varied from 1–2 episodes per day to 1 episode per 2–3 months. They had both febrile and afebrile seizures. The febrile seizures tended to evolve into SE. However, patient 6 developed permanent hemiplegia after 2 prolonged SE, which lasted about 5 h, manifesting as HHE. While patients 4 and 5 were diagnosed with DEE according to the recurrent refractory epileptic seizures and delayed development. Patient 7 presented with recurrent febrile convulsions from 1 year old and afebrile convulsions from 1 year and 7 months. No focal seizures and SE occurred. There were no patients diagnosed with EIEE, with seizure onset earlier than 6 months.

### Developmental Delay

All the patients had developmental delay in language and/or motor skills. Among them, five patients (patients 1–5) presented both motor and language developmental delay. The delayed motor milestones, with a median age of controlling the head at 9 months (ranging from 4 month to 15 month), the median age of sitting unsupported at 20 months (ranging from 8 month to 3 year), and the median age of walking independently at 4 years (ranging from 2 year 6 month to 6 year). Cognitive evaluation with the Fourth edition of Wechsler Intelligence Scale for Children was performed on three older patients (patients 1, 2, and 3). Patients 1 and 3 had mild ID with an IQ of 62 and 65, respectively at the age of 6. Patient 2 had moderate ID with an IQ of 45 at age 7 years. Patients 6, 7, and 8 only had language developmental delay. Patient 6 could pronounce 2 syllables at age 22 months. Patients 7 and 8 acquired disyllabic words at age 24 and 30 months, respectively, and spoke simple sentences at age 3 and 4 years, respectively.

With increasing age, motor development gradually improved. By the last follow-up, all patients were able to walk independently, with a minimum age of 22 months. While there was no significant improvement in intellectual development. By the time of the last follow-up, patients 1 and 3, aged 15 years and 8 years, respectively, attended special education school due to mild ID, with poor academic performance. Patient 2, aged 7 years old, did not go to school with moderate ID.

### Neuroimaging

**Figure 2:**
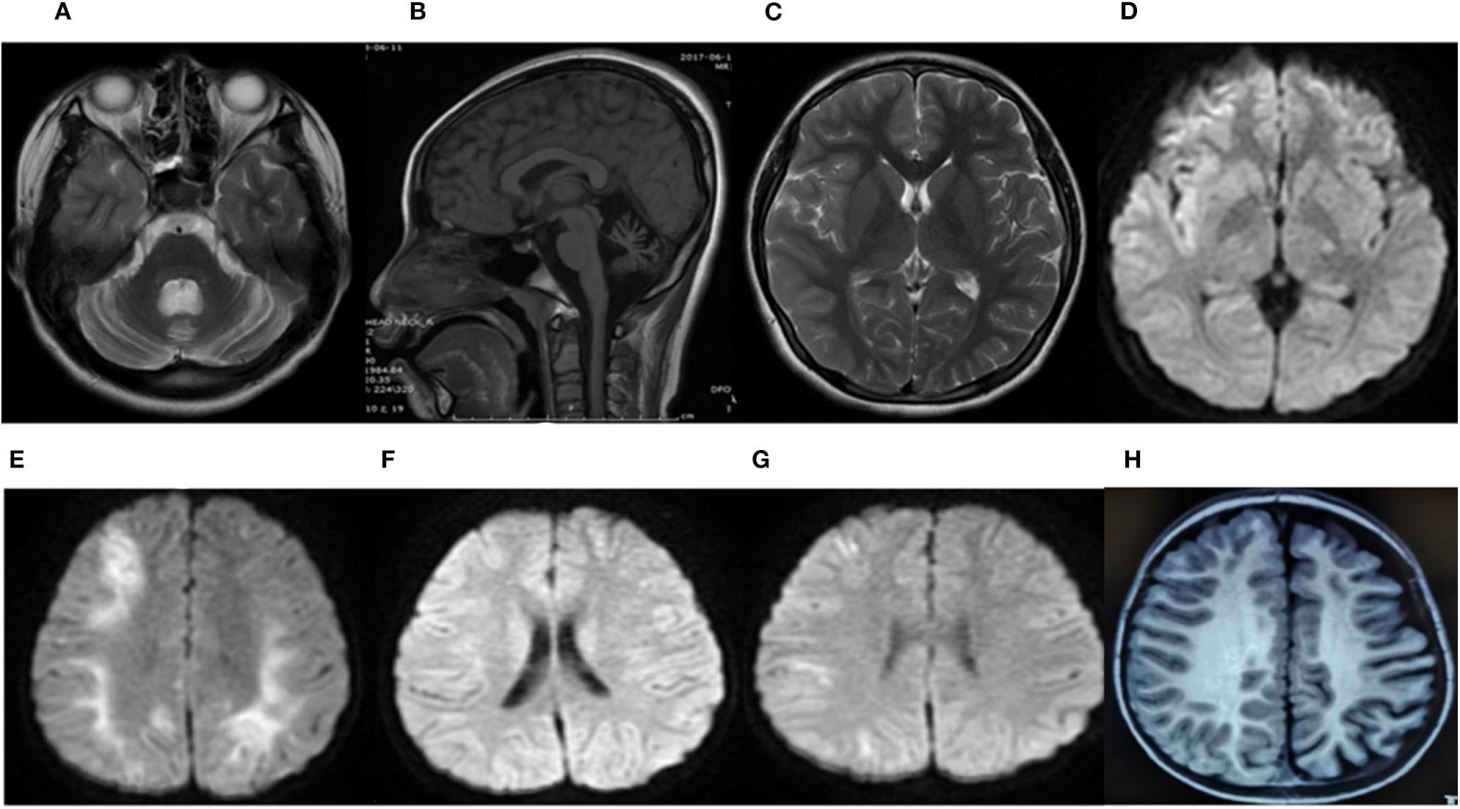
Brain MRI of patient 1 during the last attack at age 13 years **(A–D)**. An axial T2-weighted image (T2W) **(A)** and a sagittal T1-weighted image (T1W) **(B)** showed severe cerebellar atrophy. An axial T2-weighted image (T2W) **(C)** and an axial diffusion-weighted image (DWI) **(D)** showed a slight hyperintense signal in the frontal, temporal, and occipital region of the right hemisphere. The brain MRI of patient 3 **(E–G)**. The axial diffusion-weighted image (DWI) at age 3 during a severe attack **(E)**, 1 year later **(F)**, and at 6 years old soon after the last episode **(G)**. **(E)** Revealed restricted diffusion in the bilateral hemisphere. **(F)** Demonstrated a complete resolution of the cortical lesion. **(G)** Showed a mild restricted diffusion in the right hemisphere. The brain MRI of patient 6 at 22 months of age **(H)**. An axial T1-weighted image (T1W) showed significant atrophy of the left hemisphere.

Brain neuroimaging was performed on all patients. It was abnormal in four patients. In patient 1, the brain CT scan demonstrated cerebellar dysplasia at age 8 months old. During the last hemiplegic migraine attack at the age of 13, the brain MRI showed pronounced cerebellar atrophy and right hemisphere edema (Figures 2A–D). In patient 2, the brain MRI showed left hemisphere and cerebellar vermis atrophy at age 7. It was different from the MRI done at age 3, which was completely normal. In patient 3, a serial of brain MRI were performed. It revealed bilateral hemisphere edema and mild cerebellar atrophy during a severe attack at the age of 3. One year later, the MRI demonstrated the complete resolution of the cortical lesion. The third MRI performed at age of 6, soon after the last episode, showed mild focal edema in the right hemisphere (Figures 2E–G). In patient 6, the brain MRI showed significant atrophy of the left hemisphere at the age of 7 (Figure 2H).

In conclusion, cerebellar atrophy was observed in three patients (patients 1, 2, and 3). Cerebral edema was noticed in two patients during the attack period (patients 1 and 3). Hemisphere atrophy was found in two patients (patients 2 and 6).

### Video-EEG

**Figure 3:**
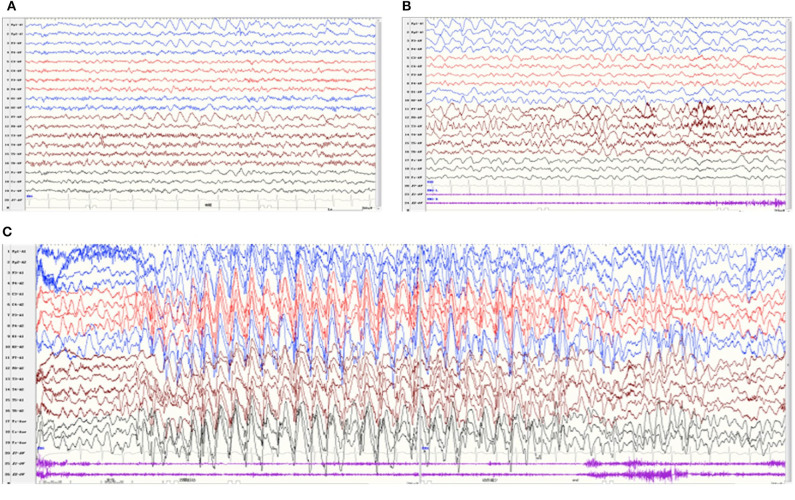
Video-EEG of patient 6 at 22 months of age. The interictal EEG revealed continuous slow theta high-amplitude activity on the left hemisphere, and rapid β-wave dominance in the right hemisphere **(A)**. The ictal EEG showed a focal discharge originating from the left temporal region **(B)**. Video-EEG of patient 7 **(C)**. The ictal EEG revealed generalized paroxysmal high-amplitude 2-3 Hz spike–wave discharges.

All patients underwent at least one Video-EEG examination. It was normal in patient 8. An asymmetric background with slow waves in one hemisphere was observed in four patients (patients 1, 2, 4, and 6). Focal discharge originating from the right/left temporal region was observed in two patients (patients 4 and 6), originating from the bilateral occipital region in one patient (patient 3), originating from multiple focal areas in one patient (patient 5). Take patient 6 as an example, the interictal EEG indicated an asymmetrical background, continuous slow theta high-amplitude activity on the left hemisphere, and rapid β-wave dominance in the right hemisphere (Figure 3A). The ictal EEG showed a focal discharge originating from the left temporal region (Figure 3B). Generalized discharge was detected in patient 7, manifesting as paroxysmal high-amplitude 2–3 Hz spike–wave discharge (Figure 3C).

### Treatment

Three patients (patients 1, 2, and 3) with hemiplegic migraines were treated with intravenous corticosteroids and IVIG, as well as antiviral drugs during the acute episodes, before *CACNA1A* gene variants were found. Patient 1 commenced her use of flunarizine at 13 years of age. She had been on this treatment for 2 years without further episodes. Patient 3 was previously diagnosed with epilepsy and treated with levetiracetam (LEV) at age 2, without definite benefit. Topiramate (TPM) was added at 6 years old, and she had not had any episodes over the past 2 years. Patient 4 was treated with LEV and oxcarbazepine (OXC) at age 15 months after two severe epileptic seizures. Right-side and left-side clonic seizures occurred at age 16 months and 21 months, respectively. TPM was added, which controlled the seizures. Patient 5 presented with hemi-clonic seizures at age 20 months. After a combined antiepileptic treatment with LEV and TPM from 2 years of age, the seizures were reduced and occurred only during fever. Patient 6 was commenced on LEV at 14 months of age without any benefit. Sodium valproate (VPA) was added at 22 months and the seizures were reduced. Patient 7 was diagnosed with epilepsy along with atypical absence seizures and tonic-clonic seizures at age 1 year and 7 months. After a combined therapy with VPA and lamotrigine (LTG), seizures had not occurred in the past 9 months. Patient 8 was diagnosed with EA2 at 4 years of age. From then on, he was treated with acetazolamide, the frequency of ataxic episodes had decreased in the past 6 months.

### Genotype-Phenotype Correlations

Seven *CACNA1A* gene variants were found in eight patients, including six missense variants and one frameshift variant. Four missense variants were in the transmembrane segment. Mutation p.R1352Q, located in the transmembrane S4 segment of Domain III was found in patients 1 and 2, both of them were diagnosed with SHM1. Missense variants of p.G701V, p.A713T, located in the transmembrane S6 segment of Domain II were found in patients 3 and 4 with different diagnosis, patient 3 was diagnosed with SHM1, whereas patient 4 was diagnosed with DEE. Mutation p.V1393M, located in the transmembrane S5 segment of Domain III was found in patient 5, who was diagnosed with DEE. The other two missense variants p.Y62C and p.F1814L, located in the cytoplasmic side of the N-terminus and C-terminus were found in patients 6 and 7, which led to HHE and epilepsy with tonic-clonic and atypical absent seizures, respectively. The only frameshift variant p.Q681Rfs^*^100, localized in the extracellular loop between S5 and S6 of Domain II, was found in patient 8 with EA2. All the seven variants of the *CACNA1A* gene were associated with ataxia and developmental delay, and all the missense variants were related to the severe epileptic seizures of SE, except one, located in the C-terminus of the gene, with tonic-clonic and atypical absent seizures controlled by antiepileptic drugs.

## Discussion

*The CACNA1A* gene encodes the voltage-dependent P/Q-type calcium channel subunit alpha-1A, which consists of four homologous regions (I–IV), each containing six transmembrane segments. The S4 segment connects to a positively charged amino acid to form the S4 transmembrane α-helix, which acts as a “voltage sensor.” The S5–S6 connecting section forms a channel hole, which selectively allows the passage of ions. The P/Q-type voltage-gate Ca_V_2.1 calcium channel is situated in the presynaptic terminal of glutamatergic and GABAergic neurons (13, 14). It is involved in the activation of postsynaptic receptors and the generation of action potential (15). Variants in the *CACNA1A* gene can lead to variable clinical phenotypes.

FHM1/SHM1 is a rare migraine with an aura subtype caused by *CACNA1A* variants, which is characterized by hemiparesis and occasionally encephalopathy during migraine attacks. A migraine with episodic hemiplegia is the core symptom. Fully reversible motor weakness is considered as the motor aura, along with at least one of the visual, sensory, aphasic, and brainstem auras (16). The migraine may be followed by the aura or may occur before the aura, its duration is uncertain, and occasionally absent. The aura lasts from several minutes to hours, and may last up to 4 weeks when hemiplegia acts as the aura (16, 17). In rare cases, diplegia has been reported as a motor aura symptom (18). Compared with FHM, SHM patients tends to have less non-motor auras, longer and more severe attacks, manifesting as acute encephalopathy, characterized by hemiplegia, coma, fever, convulsions, and so on (19). It is thought to be associated with increased glutamic acid release, decreased cortical spreading depression (CSD) threshold, and prolonged and repetitive CSD, all of which lead to cytotoxic and vasogenic edema (17, 20). In our cohort, three patients were diagnosed with SHM1, with the age of onset between 2 to 4 years, which was much earlier than that of FHM1 as reported before (17). All of them suffered severe acute encephalopathy, which was usually triggered by minor head trauma. When accompanied with fever, it often progressed to coma and prolonged hemiparesis (2 to 21 d). It is considered that the variants in the *CACNA1A* gene may act as a predisposing factor for inflammation-related encephalopathy. The increase of inflammatory factors such as IL-6 found in SHM1-related encephalopathy leads to neurogenic inflammation (21), which may account for the fact that patients with FHM1/SHM1 often presented with non-infectious fevers.

Corticosteroids theoretically mitigate CSD and edema and decrease the pain and duration of an acute HM attack (7). It has been confirmed by recent reports. After corticosteroids treatment, the severity and duration of acute attacks in the presence of encephalopathy and cerebral oedema in patients with CACNA1A variants were rapidly reduced (17). In our cohort, three patients with HM were treated with intravenous corticosteroids, and they all made a full recovery. In animal studies, the long-acting Ca^2+^-channel blocker flunarizine was suggested to block neuronal Na^+^ and Ca^2+^ currents and raise CSD thresholds, possibly decreasing cortical hyperexcitability. In our cohort, patient 1 commenced the use of flunarizine at 13 years of age. She had been on this treatment for 2 years with no further episodes. Topiramate was reported to worsen the symptoms in a single HM case (17), however, in our cohort, TPM was added to patient 3 at 6 years of age, and she has not had an episode over the past 2 years.

The P/Q channel is widely expressed in the Purkinje and granule cells of the cerebellum (22), the *CACNA1A* gene variant is closely associated with cerebellar ataxia. All the patients in our study presented with cerebellar ataxia, including one congenital ataxia, one episodic ataxia, and six non-progressive ataxia. But none of them were diagnosed with SCA6, which has a high rate of incidence in adults, whereas our patients were young. There were only three patients (patients 1–3) with SHM1 who had cerebellar atrophy in the brain MRI at age 8 months to 7 years. In patient 2, ocular strabismus and hypotonia were observed at 3 months after birth, whereas, the brain MRI did not show cerebellar atrophy until 7 years old. In 2019, Humbertclaude V et al. reported that 5 out of 18 patients with *CACNA1A* variants had cerebellar atrophy in MRI, and patients with abnormal MRI tended to be older than 10 years (2). These findings indicate a functional cerebellar defect before the onset of the neurodegenerative process or a low sensitivity of conventional neuroimaging in the diagnosis of early cerebellar damage (23).

Variants in *CACNA1A* may also cause episodic or persistent tremors. In 2016, Jiang et al. reported a 19-year-old boy with a *CACNA1A* gene variant that had recurrent attacks of head and trunk tremors, intentional tremors, and signs of cerebellar ataxia (24). Static tremors have been reported in patients with EIEE caused by *CACNA1A* variants (5, 9). In our cohort, two patients (patients 4 and 5) had static tremors in the head and trunk. Paroxysmal events such as BPV, BPT, and PTU have also been reported. PTU was noticed in patient 2 at age 4 months before the attacks of HM.

Seizures/epilepsy is a common symptom in S/FHM1, the penetrance of epilepsy is higher compared to S/FHM2 and S/FHM3 (25). In our cohort, seizures/epilepsy occurred during HM attacks in all three SHM1 patients, and between migraine attacks in patient 3. Indeed, epilepsy is a very common symptom of *CACNA1A* gene variants beside FHM1/SHM1. EIEE 42 has recently been reported in patients with *CACNA1A* variants. It occurs in early infancy and is frequently characterized by myoclonic, tonic, and tonic-clonic attacks, which act as generalized seizures with a general discharge in an EEG (8, 26). In this cohort, none of the patients met the diagnostic criteria of EIEE. Seven out of the eight patients developed seizures with an onset later than 6 months, in which only one patient exhibited atypical absence seizures and generalized tonic-clonic seizures, while others exhibited focal seizures.

In general, ion channel genes such as the *SCN1A, SCN1B*, and *GABRB2* variants tend to develop seizures with heat sensitivity. The *CACNA1A* variant has the same characteristic, and is prone to SE, while attacks in clusters are rare. SE probably shares a similar pathogenesis with HM-related encephalopathy (27). Both patients with focal SE (patients 4 and 6) and patients with HM-related encephalopathy (patients 1 and 2) displayed an asymmetric background EEG rhythm with slow waves in one hemisphere, which were associated with cerebral edema in the hemisphere due to repeated CSD activity. In our cohort, seven patients presented with heat-sensitive epileptic seizures. The seizures in most of the patients were severe, five suffered from SE and one (patient 6) of them developed permanent hemiplegia after two prolonged SE, manifesting as HHE, with contralateral cerebral hemisphere atrophy of the hemiplegia in the brain MRI, while patient 3 did not develop HHE after several SE attacks, with reversible brain edema in MRI. Nadine Pelzer et al. indicated that repeated CSD can lead to severe cytotoxic edema, some of which is reversible, however, some edema is irreversible and eventually leads to the death of neurons, which manifest as brain atrophy in neuroimaging and probably HHE in the clinical setting (20).

In 2019, 18 children with *CACNA1A* mutations were reported, and 9 out of 18 patients presented with cognitive impairment, including developmental delay, cognitive difficulties, and learning difficulties (2). All the eight patients reported here had developmental delay in language and/or motor skills. ID was found in three older patients with SHM1 and cerebellar ataxia, ranging from mild to moderate intellectual impairment. Cerebellar atrophy was shown in the brain MRI. It was assumed that patients with vermian atrophy were at a higher risk of cognitive dysfunction (2). The development delay of a child also suggested an impairment of cognitive networks due, besides the cerebellar dysfunction, also to an abnormal hippocampal neurotransmission as documented in a knock-in mice model expressing the FHM1 mutation (28). With increasing age, motor development was gradually improved, while no significant improvement was seen in intellectual development. It indicated that the cognitive impairment caused by *CACNA1A* mutations was usually persistent.

The phenotypes are usually overlapping. Cerebellar ataxia is a common phenotype considering the damage caused by the *CACNA1A* variant to cerebellar function and/or structure. Other phenotypes, including HM, epilepsy, and EIEE, have been reported on this basis (5, 29, 30). In addition, cognitive dysfunction and/or developmental delay are also often noted due to the relationship between cerebellar dysfunction and cognitive impairment. The functions of the posterior cerebellar vermis and flocculus are impaired early in patients with *CACNA1A* variants, accounting for the manifestation of the paroxysmal events in infancy, which may be the early manifestation of *CACNA1A* variants in childhood (31). With increasing age, new phenotypes appear in succession. However, whether there is an age-dependent effect on phenotype is unclear.

The phenotype was partly related to the genotype. The *CACNA1A* gain-of-function (GOF) mutations could enhance the neuronal excitability by increasing the influx of calcium ions and release of glutamate, decreasing the threshold of CSD, resulting in the occurrence of HM and epilepsy (32). The *CACNA1A* loss-of-function (LOF) mutations induce the synaptic dysfunction of cortical interneurons and specifically reduce or impair the function of cortical GABA neurotransmitters, resulting in the occurrence of EA2 and epilepsy (33). Both GOF and LOF *de novo* missense variants caused severe developmental epileptic encephalopathies (DEEs) (9). While SCA6 is caused by the expansion of a polyglutamine repeat in the intracellular C-terminus of the α1 subunit of the P/Q channel, leading to toxic accumulation of polyQ and subsequent selective degeneration of the cerebellar Purkinje cells (5).

FHM1/SHM1 is associated with *CACNA1A* GOF mutations, most of which are missense variants. In our series, patients 1 and 2 harbored the same *de novo* missense variant, p.R1352Q (also referred as p.R1349Q in NM-001127221), which were reported previously. Patients harboring this variant usually manifest as SHM with recurrent episodes of coma, congenial ataxia, and developmental delay (2, 5–7), just like the manifestation of the two patients in this cohort. They did not exhibit heterogeneous phenotypes. It was confirmed in animal experiments that this variant is a GOF mutation and shifts Cav2.1 channel activation to lower voltages by ~ 6–12 mV (34). Patient 3 harbored a novel missense variant, p.G701V, which is conserved across species and may be a GOF mutation. Missense variants in nearby residues (I712V, A713T, V714A, and D715E) have been reported in the Human Gene Mutation Database in association with FHM and EIEE (12, 34). The missense variants in patients with F/SHME1 (F/SHM1 and epilepsy) lay mostly in the III and IV S4, S5, or S6 transmembrane domains (25). In our cohort, patients 1 and 2 had the mutation p.R1352Q located in the transmembrane segment S4 of domain III, and patient 3 had the mutation p.G701V located in the transmembrane segment S6 of domain II.

Disrupting variants that result in a loss of function of the Cav2.1 calcium channel are the most commonly reported changes in EA2 patients. Patient 8 with EA2 harbored p.Q681Rfs^*^100, the only frameshift variant in this study, which caused a premature stop codon 100 codons downstream. The variant led to a truncated protein and resulted in the loss of function of the Cav2.1 calcium channel (4, 35).

Both GOF and LOF mutations could cause epileptic seizures. The mutations C.2137G > A (p.A713T) and C.4177G > T (p.V1393M) detected in patients 4 and 5 had been reported previously in patients with EIEE. They are located in the transmembrane S6 segment of Domain II, and S5 segment of Domain III, respectively. Both of them have been proven to be GOF missense mutations by a previous molecular electrophysiological study, causing DEE with severe refractory seizures starting in the first 6 months of life, global developmental delay evolving toward moderate to severe ID, and variable motor symptoms (ataxia, tremors, hypotonia) (5, 9). Patients 4 and 5 in this cohort manifested with DEE and tremors, whereas the onset age was later than 6 months. It indicated that patients with the same variation may have a different age of onset. Other modifier genes and/or environmental factors may play a regulator role in the phenotypic variability. Patient 6 had a novel missense variant, C.185A > G (p.Y62C), which was the first missense variant found in the N-terminus. Patient 7 harbored a novel missense variant, C.5442T > G (p.F1814L), located in the beginning of the cytoplasmic C-terminus, which is highly conserved. The adjacent variant, p.I1811L, had been reported as a GOF mutation in two unrelated FHM families (36). While the R1820 stop mutation was identified in a patient with frequent absence attacks, which was similar to our patient. The variant was confirmed as a LOF mutation which substantially impaired the channel function (37). The N-terminus and C-terminus of the α1 subunit have important roles in trafficking and anchoring the subunit to the cell membrane (38). The symptoms of recurrent SE and permanent hemiplegia caused by the missense variant, p.Y62C in the N-terminus, were more severe than that caused by the variant (p.F1814L) in the C-terminus.

In conclusion, *CACNA1A* mutations can lead to a wide spectrum of neurological disorders. The symptoms usually overlap with the common presentation of episodic neurological manifestations (epileptic or non-epileptic paroxysmal events, epileptic or non-epileptic encephalopathy), and chronic symptoms (cerebellum ataxia and developmental delay). It tends to develop seizures with heat sensitivity and is prone to SE. Both GOF and LOF mutations were identified in *CACNA1A*-related disorders. Four *de novo* missense mutations were found in five patients located in the S4, S5, or S6 transmembrane segments of Domains II and III (p.R1352Q, p.G701V, p.A713T, p.V1393M). All of them were correlated with severe phenotypes. However, the relationship between genotype and phenotype is unclear, it needs to be confirmed in larger studies in view of the limited number of patients in our cohort.

## Data Availability Statement

The datasets presented in this study can be found in online repositories. The names of the repository/repositories and accession number(s) can be found in the article/supplementary material.

## Ethics Statement

This study was approved by the Clinical Research Ethics Committee, Peking University (No. [2005] 004). Written informed consent was obtained from parents of participants or participants of the study.

## Author Contributions

XB designed and conceptualized the study and revised the manuscript. LZ designed the study, collected patient information, and drafted the manuscript. YW provided help in the genetic analysis. QZ, YC, JW, KS, and LD provided help in the collection of patient. All authors contributed to the article and approved the submitted version.

## Conflict of Interest

The authors declare that the research was conducted in the absence of any commercial or financial relationships that could be construed as a potential conflict of interest.
